# Molecular mechanisms for the evolution of bacterial morphologies and growth modes

**DOI:** 10.3389/fmicb.2015.00580

**Published:** 2015-06-09

**Authors:** Amelia M. Randich, Yves V. Brun

**Affiliations:** Department of Biology, Indiana University, Bloomington, IN, USA

**Keywords:** bacterial shape, bacterial morphology, peptidoglycan synthesis, *Caulobacter*, *Asticcacaulis*, FtsZ, MreB

## Abstract

Bacteria exhibit a rich diversity of morphologies. Within this diversity, there is a uniformity of shape for each species that is replicated faithfully each generation, suggesting that bacterial shape is as selectable as any other biochemical adaptation. We describe the spatiotemporal mechanisms that target peptidoglycan synthesis to different subcellular zones to generate the rod-shape of model organisms *Escherichia coli* and *Bacillus subtilis*. We then demonstrate, using the related genera *Caulobacter* and *Asticcacaulis* as examples, how the modularity of the core components of the peptidoglycan synthesis machinery permits repositioning of the machinery to achieve different growth modes and morphologies. Finally, we highlight cases in which the mechanisms that underlie morphological evolution are beginning to be understood, and how they depend upon the expansion and diversification of the core components of the peptidoglycan synthesis machinery.

## Introduction

**FIGURE 1:**
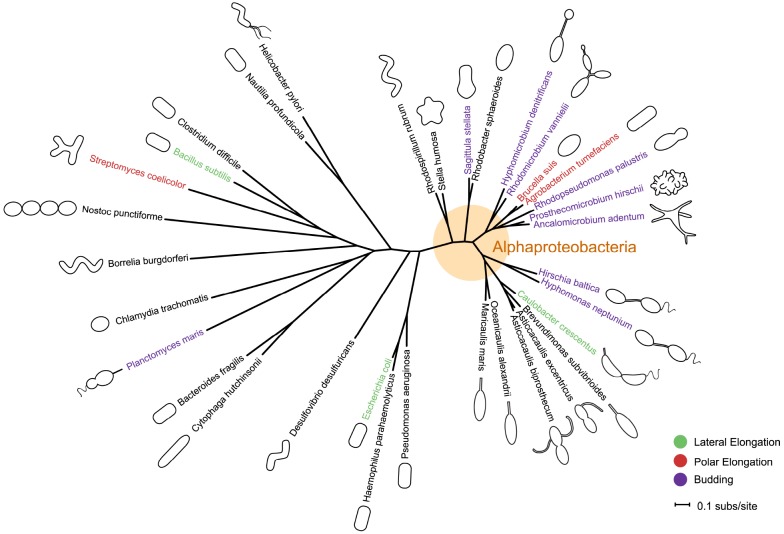
**Phylogeny and morphology of bacteria exhibiting different modes of growth.** *rpoC* sequences were aligned using MUSCLE v3.8.31 (Edgar, 2004). RAxML v7.0.4 (Stamatakis, 2006) reconstructed the maximum likelihood phylogeny using the JTT amino acid substitution matrix with rate variation among sites modeled by a four-category discrete gamma distribution and an additional invariant category. Note that the stalk of *Planctomyces maris* is composed of several parallel fibrils (Bauld and Staley, 1976), and is not bound by cell envelop layers like that of the Caulobacterales. Bacteria are not drawn to scale. Scale bar reflects numbers of substitutions per site.

The typical of bacterial shapes as simply rods, variations of rods, or cocci belies the great diversity of bacterial morphologies. Within these simple classifications, bacteria exhibit a broad range of morphologies—helical or vibrioid twists, filaments, hyphae or branched filaments, prosthecae or stalks (thin cylindrical extensions of the cell envelope)—as well as distinct growth modes, forms of cellular differentiation, and life cycles (Figure 1, Young, 2006). The mechanisms by which bacteria achieve these morphologies, much less evolve them, has remained unclear. Historically, the murein sacculus, or peptidoglycan layer, has been posited as the underlying determinant of bacterial shape (Vollmer et al., 2008a). A heteropolymer of peptide cross-linked glycan strands, the sacculus confers strength to the cell wall and maintains cell shape and size. It is becoming clear that the sacculus in and of itself is not the only determinant of cell shape; rather it is the spatiotemporal regulation of the enzymes that build the sacculus and modify it in response to environmental changes that drives morphogenesis (Young, 2010; Cava and de Pedro, 2014).

**FIGURE 2:**
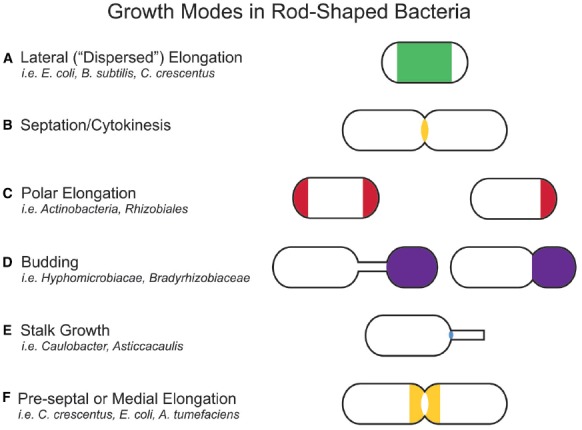
**Growth modes in rod-shaped bacteria.** Several growth modes with the regions of active peptidoglycan synthesis are schematized. Colors indicate regions of active peptidoglycan synthesis. **(A)** Lateral elongation is well-studied in *E. coli*, *B. subtilis*, and *C. crescentus*, and often assumed for many rod-shaped bacteria. **(B)** A majority of bacterial families divide in a FtsZ-dependent manner. Notable exceptions are members of the PVC (Planctomycetes, Verrucomicrobia, and Chlamydiae) superfamily. **(C)** Polar elongation is characteristic of Actinobacteria, where it is bipolar. It has recently been demonstrated in Rhizobiales (Brown et al., 2012) where it is unipolar. **(D)** Budding occurs both at the ends of stalks and off the cell body in Alphaproteobacteria. **(E)** Stalks are wide-spread in Alphaproteobacteria. While the exact mechanism for growth may differ among families, all depend on zonal growth of the peptidoglycan at the cell-stalk junction. **(F)** Some species exhibit medial (pre-septal) growth, which appears to be peptidoglycan synthesis near the division plane before full assembly of the Z-ring.

In bacteria, morphologies and growth modes are interconnected (Brown et al., 2011). In many taxa, maintenance of the rod shape itself requires at least two well-studied modes of growth: lateral elongation (Figure 2A), or incorporation of peptidoglycan along the sidewalls; and septation (Figure 2B), the generation of nascent poles, usually at the cell center. In these canonical cases, which are exemplified by model organisms *Escherichia coli* and *Bacillus subtilis*, material at the poles remains inert, with no evidence of new peptidoglycan incorporation or turnover (Mobley et al., 1984; Schlaeppi et al., 1985; de Pedro et al., 1997; Janakiraman and Goldberg, 2004). However, other, less understood approaches to achieving rod-shaped morphologies exist. Actinobacteria exhibit polar growth (Figure 2C), where elongation occurs strictly at one or both poles, leaving the sidewalls, instead of the poles, inert (Umeda and Amako, 1983; Daniel and Errington, 2003; Chauhan et al., 2006; Flärdh and Buttner, 2009; Flärdh, 2010). Alphaproteobacteria (the superfamily highlighted in Figure 1), exhibit a diverse mix of growth strategies including lateral elongation and polar growth, but also the unknown mechanisms of zonal growth to produce buds and/or stalks (Figures 2D,E; Brown et al., 2011). In a striking example, bacteria in the family *Hyphomicrobiaceae* not only produce a stalk, but also bud daughter cells from the end of the stalk (Figure 2D) via an unknown mechanism (Whittenbury and Dow, 1977; Moore, 1981). The life cycle of these cells involves at least three separate modes of growth: stalk elongation at the junction of the cell body and stalk, daughter cell elongation at the tip of the stalk, and septum formation within the stalk to complete division (Moore and Hirsch, 1973; Whittenbury and Dow, 1977; Moore, 1981).

In comparison to lateral elongation and septation, much less is known genetically or mechanistically about polar growth or budding, making it hard to rigorously distinguish between these growth modes. The next section will describe how, although lateral elongation and septation differ at the genetic level, they ultimately drive distinct growth modes by positioning functionally similar peptidoglycan synthesis machineries at specific locations in the cell. It is likely that polar growth, budding, and stalk growth also result from the repositioning of yet to be described peptidoglycan synthesis machineries. In this way, all growth modes can be described as zonal growth (Brown et al., 2011). Already with these few examples, a great plurality of growth modes clearly has evolved to generate a “common” rod shape, as well as to achieve novel morphologies and life cycles. This review aims to summarize the spatiotemporal mechanisms that target peptidoglycan synthesis to different subcellular zones to generate the rod-shape of model organisms *E. coli* and *B. subtilis* and to then describe how the modularity of the core components of the peptidoglycan synthesis machinery permits repositioning of the growth machinery to achieve different growth modes and morphologies. Because of space limitations, we were unable to cover the septal elongation mechanisms that generate round and ovoid cells and we refer the reader to excellent recent reviews on this topic (Sham et al., 2012; Pinho et al., 2013).

## Molecular Machinery of Bacterial Growth and Division in Rod-shaped Bacteria

The molecular underpinnings of how bacteria grow, divide, and maintain shape are slowly emerging, especially in model rod-shaped bacteria such as *E. coli* and *B. subtilis*. In these species, two protein assemblies direct the modification and synthesis of the sacculus at specific times and locations during the cell cycle: the elongasome inserts new peptidoglycan along the length of the rod during growth, and the divisome completes the steps of constriction and new peptidoglycan synthesis at the cell center during cell division (Typas et al., 2012). Both of these assemblies utilize similar components and likely share a common evolutionary history (Szwedziak and Löwe, 2013). The preponderance of conserved protein classes for peptidoglycan synthesis machinery across gram-negative and positive bacteria, as well as between the elongasome and divisome assemblies, suggests a general strategy for shaping the bacterial cell as well as molecular mechanisms for the evolution of novel morphologies.

**FIGURE 3:**
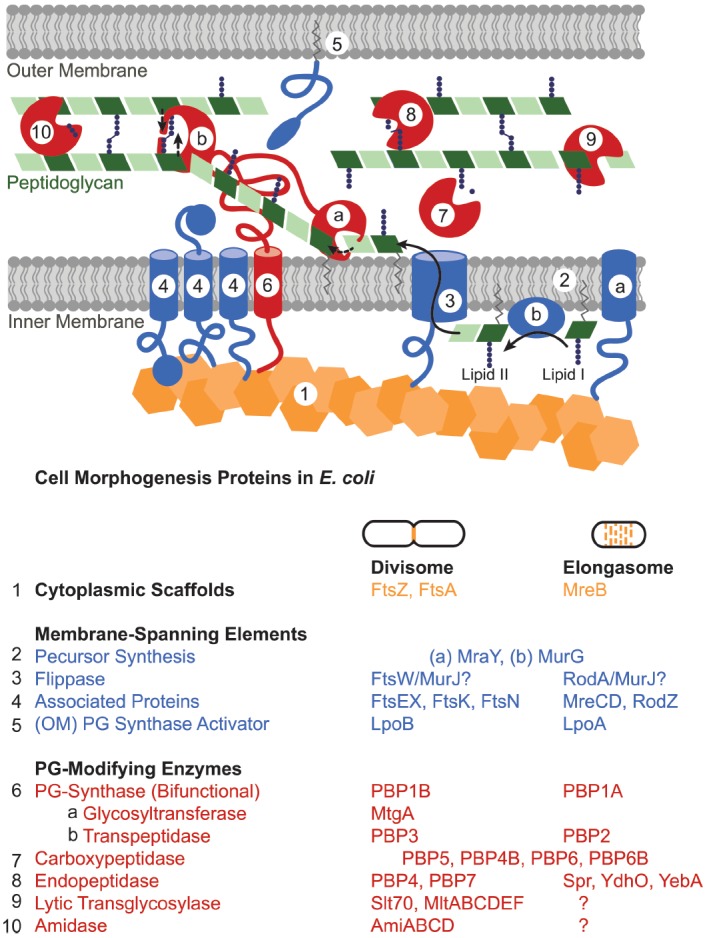
**Peptidoglycan synthesis machinery schematic.** The divisome and elongasome both consist of cytoplasmic scaffolds (orange), membrane-spanning elements that include regulatory proteins as well as peptidoglycan precursor synthesis machinery (blue), and peptidoglycan-modifying enzymes (red). Each assembly has genetic components that are biochemically distinct despite retaining similar functions (Table). In general, (1) cytoplasmic scaffolds such as FtsZ or MreB direct the location of peptidoglycan synthesis and recruit various cytoplasmic and inner-membrane components. (2) Cytoplasmic and inner membrane enzymes synthesize peptidoglycan monomers (lipid II) in the cytoplasm [only (a) MraY and (b) MurG shown] and (3) a flippase flips them across the inner membrane. (4, 5) Various membrane proteins function to regulate or organize the divisome or elongasome protein assemblies. (6) Bifunctional or monofunctional peptidoglycan synthases (a) polymerize lipid II into glycan strands and (b) crosslink the peptide chains to form the sacculus. Various enzymes modify the peptidoglycan after synthesis: (7) carboxypeptidases trim peptide chains, (8) endopeptidases cleave crosslinks, (9) lytic transglycosylases cleave the glycan strand, (10) and amidases remove peptide chains from the glycan strand. Question marks in the table indicate that elongasome proteins responsible for the indicated activities remain to be identified. Some proteins essential to the divisome have been omitted: ZipA, ZapABCD, FtsQ, FtsL, and FtsB.

The divisome and elongasome have long been hypothesized to form large complexes (Höltje, 1998). Both consist of cytoplasmic scaffolding cytoskeletal-like proteins; inner membrane-spanning elements; and a host of periplasmic enzymes including peptidoglycan synthases and hydrolases (Figure 3; Margolin, 2009; Typas et al., 2012; Egan and Vollmer, 2013). This machinery works in concert to create the sacculus, a meshwork consisting of chains of two alternating sugar types, *N*-acetyl glucosamine and *N*-acetyl muramic acid, joined through beta-(1,4)-glycosidic bonds that are cross-linked together via peptide chains. The steps in peptidoglycan synthesis proceed as follows: a series of enzymes generates a pool of nucleotide precursors in the cytoplasm, a flippase then transfers the lipidated monomers over the inner membrane, and finally, peptidoglycan synthases polymerize the monomers into glycan chains and cross-link them through peptide bonds, thus forming the sacculus. Other peptidoglycan-modifying enzymes further diversify the periplasmic/extracellular components, such as the carboxypeptidases that trim peptide chains and the lytic transglycosylases that reduce the length of glycan strands. Moreover, peptidoglycan hydrolases, which cleave either the glycosidic or amide bonds of peptidoglycan, play important roles in turning over old peptidoglycan to allow insertion of new material in the growing cell and help shape the new poles (Priyadarshini et al., 2007; Vollmer et al., 2008b; Frirdich and Gaynor, 2013; Lee and Huang, 2013).

The cytoplasmic scaffold FtsZ organizes and regulates the activity and localization of various divisome components. Much progress has been made in elucidating how the divisome is spatiotemporally regulated in canonical rod-shaped bacteria. During division, the GTP-dependent polymerization of the tubulin-like FtsZ creates a ring-shaped structure called the Z ring at the cytoplasmic face of the membrane at the center of the cell (Bi and Lutkenhaus, 1991; Löwe and Amos, 1998; Mukherjee and Lutkenhaus, 1998). The exact architecture of the Z-ring has recently been the subject of intense study (Li et al., 2007; Fu et al., 2010; Strauss et al., 2012; Szwedziak et al., 2014). Once assembled, the Z-ring recruits over 10 essential divisome components (Egan and Vollmer, 2013). In *E. coli*, the mechanism for targeting the Z-ring to the center of the cell involves the well-characterized Min system, which prevents Z-ring assembly at the cell poles (Raskin and de Boer, 1999; Hale et al., 2001), and nucleoid occlusion protein SlmA (Bernhardt and de Boer, 2005). *B. subtilis* appears to maintain many of the same essential division proteins with the exception of utilizing DivIVA to regulate the septation site by positioning and stabilizing Min proteins at the cell poles (Edwards and Errington, 1997; Marston et al., 1998; Hamoen and Errington, 2003) and using a nucleoid occlusion mechanism mediated by a different protein, Noc (Wu and Errington, 2004). Other species also utilize different systems for positioning FtsZ, such as the MipZ system in *Caulobacter crescentus* (Thanbichler and Shapiro, 2006), the MapZ/LocZ system in *Streptococcus pneumoniae* (Fleurie et al., 2014; Holečková et al., 2015), and the SsgB system in *Streptomyces* (Willemse et al., 2011). Each of these systems (with the exception of SsgB) faithfully localizes the Z ring to the division plane with high precision at onset of division (Trueba, 1982; Yu and Margolin, 1999; Männik et al., 2012).

Much less is known about the spatiotemporal regulation of the elongasome, for which MreB appears to be the major scaffold for coordinating peptidoglycan precursor synthesis and peptidoglycan polymerization. MreB is an actin-like protein that forms membrane-associated filaments (Esue et al., 2005, 2006; Salje et al., 2011; Ozyamak et al., 2013). Once thought to form a cytoskeletal meshwork, MreB has since been shown to form discrete, motile patches that move, independently of MreB polymerization or treadmilling, near-perpendicularly to the long cell axis in *E. coli*, *B. subtilis*, and *C. crescentus* (Dominguez-Escobar et al., 2011; Garner et al., 2011; van Teeffelen et al., 2011). MreB interacts with inner membrane proteins MreC, MreD, RodZ (Kruse et al., 2004; van den Ent et al., 2006; Shiomi et al., 2008; Alyahya et al., 2009; Bendezú et al., 2009), as well as lipid II synthesis enzymes MraY and MurG (Mohammadi et al., 2007), and its movement during elongation depends on both the synthesis of essential peptidoglycan components and the activity of peptidoglycan synthases (Dominguez-Escobar et al., 2011; Garner et al., 2011; van Teeffelen et al., 2011). Other components of the elongasome, such as MreB-associated proteins MreCD and certain PBPs, have been shown to exhibit the same motile, spatiotemporal localization as MreB (Dominguez-Escobar et al., 2011; Garner et al., 2011), suggesting that parts of the elongasome indeed travel as a complex. MreB could function to coordinate elongasome complexes and perhaps restrict their mobility to ensure a uniform distribution of peptidoglycan insertion. Recent work combining time-lapse and 3D-imaging with computational analysis indicates that MreB preferentially localizes to and directs peptidoglycan synthesis at regions of negative curvature in *E. coli* cells (Ursell et al., 2014). Therefore MreB could selectively drive peptidoglycan growth away from the positive curvature of the cell poles to straighten the cell and create the rod shape. Possibly, other factors remain to be discovered that could regulate the localization and dynamics of MreB to in turn direct the activity of the elongasome. For example, as will be covered in the next sections, MreB interacts with *Caulobacter crescentus*-specific genes TipN and CreS.

While both the divisome and elongasome depend on similar molecular components, they clearly drive disparate morphological changes: cutting the cell in half versus lengthening the cell. These particular growth modes are achieved by the distinct localization patterns of their respective cytoplasmic scaffolds. During division, FtsZ forms a ring at the site of septation and thereby directs peptidoglycan growth and hydrolysis to shape the new poles of the daughter cells. In contrast, MreB distributes along the sidewalls of the cell to drive homogeneous insertion of new peptidoglycan in the lengthening cell. While these assemblies appear to share a similar strategies for growth, their respective components make them biochemically distinguishable (Table in Figure 3) and allow for independent regulation and incorporation into specific cell events. This modularity—evident here in the cytoplasmic scaffolds—permits wholesale repositioning of the growth machinery to achieve different growth modes and morphologies.

## Mechanisms for Evolving Novel Morphologies and Growth Modes

One can imagine multiple ways in which complexes such as the elongasome and divisome could be repurposed or retooled to evolve new morphologies and modes of growth. Theoretically, expansion and diversification of any of the core elements of the peptidoglycan synthesis assembly—cytoskeletal-like proteins, inner membrane-spanning elements, periplasmic or extracellular enzymes and outer membrane proteins—present opportunities for such evolution. The spatiotemporal interdependency of peptidoglycan synthesis machinery components in both the elongasome and divisome suggests that new relationships can evolve between different components. Moreover, the addition of new regulatory or recruitment components to the complexes may drive species-specific morphologies. Recent studies in non-canonical rod-shaped bacteria indicate that different modes of growth depend upon the same core machinery, but that these are organized or regulated differently by species-specific components.

A large diversity of bacterial cytoskeletal elements appears to underlie species-specific lifestyles and morphologies. Besides FtsZ and MreB, other self-oligomerizing protein scaffolds have been identified and shown to be widespread in bacteria (Bagchi et al., 2008; Kühn et al., 2010). In many cases, these scaffolds have roles in driving morphology. For example, intermediate filament-like crescentin (CreS) forms a single filamentous structure in *Caulobacter crescentus* that produces the characteristic vibrioid shape of the bacterium by inducing differential growth of the sides of the rod-shaped sacculus (Ausmees et al., 2003; Cabeen et al., 2009). In contrast, *Helicobacter pylori* utilizes a family of coiled-coil rich proteins (Ccrp) that form extended filamentous structures to maintain a helical rod shape (Waidner et al., 2009; Specht et al., 2011). These examples demonstrate how two scaffolding proteins, unrelated in sequence, have arisen in two different species to create twists in the rod shape. In contrast to the eukaryotic cytoskeleton, in which classes of scaffolding proteins such as actin or microtubules have been adapted to suit multiple processes by using adaptor proteins, it appears bacteria may have developed larger numbers of species- and process-specific scaffolding proteins (Ozyamak et al., 2013).

Although FtsZ and MreB comprise the core of the canonical divisome and elongasome, there are many cases in which their roles have changed or they have been eliminated from genomes completely. For example, MreB plays a role in chromosome segregation but not growth in *H. pylori* (Waidner et al., 2009), and is absent in the majority of Actinobacteria, some Firmicutes, and a minority of Proteobacteria (Margolin, 2009; Brown et al., 2011; Jiang et al., 2015). It is tantalizing to infer that MreB delineates specific growth modes since bacteria lacking MreB, such as Actinobacteria and many Alphaproteobacteria—*Agrobacterium*, *Hyphomicrobium*, and *Rhizobium*—utilize polar growth mechanisms (Brown et al., 2012, 2011). However, the story appears to be much more complex than associating MreB with lateral elongation of rod-shaped bacteria. Members of the *Chlamydiae* family represent an interesting family of bacteria that lack FtsZ while maintaining a nearly complete suite of other peptidoglycan synthesis machinery components, including MreB (Stephens et al., 1998; McCoy and Maurelli, 2006; Pilhofer et al., 2008; Bertelli et al., 2010). This is in contrast to the great majority of bacterial phyla that utilize FtsZ for cell division (Erickson et al., 2010). In fact, the presence of peptidoglycan in the *Chlamydiae* cell wall was long debated and has only recently been demonstrated in several members of the family (Pilhofer et al., 2013; Liechti et al., 2014). Localization studies in *Waddlia chondrophila* suggest that MreB acts in lieu of FtsZ to facilitate division in this species (Jacquier et al., 2014). Other studies have demonstrated that various components of the peptidoglycan synthesis pathway from FtsZ-independent species function *in vitro* and in complementation studies (Henrichfreise et al., 2009; Frandi et al., 2014). Therefore, even in genera that have diverged greatly, the overarching mechanism of peptidoglycan synthesis remains.

The expansion of various classes of peptidoglycan-modifying enzymes is becoming a common theme in studies of alternative morphologies and growth modes as less studied genera come to the forefront. *H. pylori* appears to employ a diverse cast of endo- and carboxypeptidases that likely shape the helical sacculus through alternative cross-linking (Bonis et al., 2010; Sycuro et al., 2010, 2012, 2013). In *Agrobacterium tumefaciens*, which has been recently shown to grow polarly (Brown et al., 2012), polar growth appears to depend on a class of alternative L,D-transpeptidases (Cameron et al., 2014). Overall, the modularity of the common core components of the peptidoglycan synthesis machinery provides ample flexibility for new growth modes and morphologies. Although some bacterial families have clearly shifted their dependence from central components such as MreB or FtsZ to other scaffolds, the general mechanism for assembling core peptidoglycan synthesis machineries appears to remain intact.

## Cell Growth and Morphology of *Caulobacter crescentus*

The dimorphic alphaproteobacterium *Caulobacter crescentus* represents an excellent case study in understanding how new morphologies arise from the diversification of the common core peptidoglycan synthesis machinery. *C. crescentus* divides asymmetrically, producing a swarmer cell with a polar flagellum and a DNA replication-competent cell with a polar stalk. This process of division requires strict coordination in time and space with other cell cycle events such as cell growth, chromosome segregation, and differentiation. Therefore it is unsurprising that although *C. crescentus* maintains similar core peptidoglycan synthesis machinery components in its divisome and elongasome, it has evolved discrete scaffolds, regulatory proteins, and cell cycle control mechanisms to adapt the machinery to its specific lifestyle. Determining exactly how *C. crescentus* has repurposed conserved peptidoglycan synthesis components for elongation, division, and stalk synthesis will help to expand our understanding of bacterial morphology.

**FIGURE 4:**
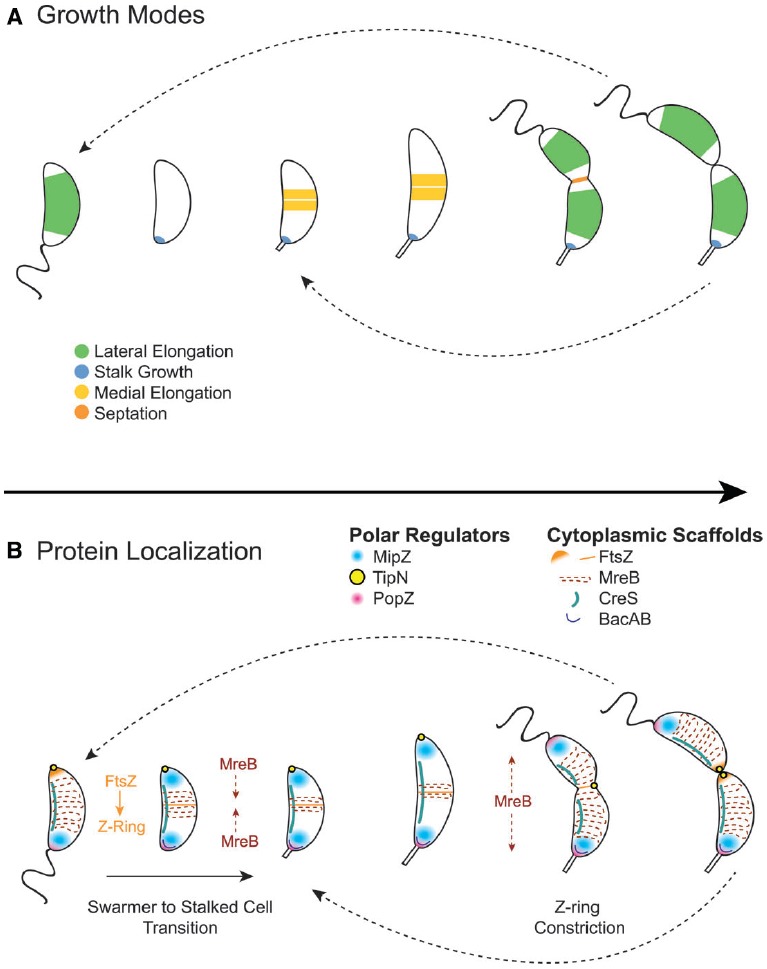
**Cytoplasmic scaffold localization dynamics and growth modes in *Caulobacter crescentus*. (A)** *C. crescentus* exhibits four growth modes over the course of its dimorphic cell cycle: lateral elongation (swarmer and stalked cells), stalk synthesis and elongation (stalked cells), medial elongation (stalked cells), and septation. Colors indicate regions of active peptidoglycan synthesis. **(B)** The activity and dynamics of various peptidoglycan synthesis machineries, which are regulated by polar regulators, underlie the different growth modes. In swarmer cells, MipZ localizes at the flagellar pole, driving FtsZ to the opposite pole, and MreB is dispersed along the length of the cell. During the swarmer to stalked cell transition, the flagellar pole undergoes remodeling to become the stalked pole. This transition involves the recruitment of bactofilin and peptidoglycan synthesis machinery to the stalked pole by the PopZ network to initiate stalk synthesis. During initiation of chromosome replication and origin duplication, some of the MipZ-ParB complex binds the new origin and migrates with it to the new pole, displacing FtsZ from the new pole to the division plane, where it forms the Z-ring. MreB condenses at the division plane before dispersing again during constriction due to regulation by TipN. After septation, FtsZ reassembles into the Z-ring quickly in stalked cells, making medial elongation the primary mode of elongation in this morphology.

The diversification of proteins interacting with and regulating FtsZ and MreB allows for strict control of asymmetric division and morphological development in *C. crescentus*. One significant difference between *C. crescentus* and the canonical rod-shaped bacteria *E. coli* and *B. subtilis* is that *C. crescentus* does not utilize a Min system or nucleoid occlusion proteins. Instead, *C. crescentus* uses MipZ gradients to position the FtsZ ring at the division plane (Thanbichler and Shapiro, 2006). MipZ directly interacts with FtsZ to inhibit ring polymerization. When MipZ associates with ParB at the stalk pole prior to S phase, it drives FtsZ monomers to the new, non-stalked pole (Figure 4B). During initiation of chromosome replication and origin duplication, some of the MipZ-ParB complex binds the new origin and migrates with it to the new pole, displacing FtsZ from the new pole to the division plane. Although MipZ shares some distant domain similarity with MinD, it likely evolved from ParA-like DNA partitioning proteins and is conserved amongst all Alphaproteobacteria without MinCD orthologues (Thanbichler and Shapiro, 2006).

Other polar regulators have been shown to influence cell morphology and interact with peptidoglycan synthesis machinery (Figure 4B). TipN, a polytopic membrane protein with a large coiled-coil cytoplasmic domain, ensures transmission of cell polarity to the daughter cell by marking the new pole and regulating MreB dynamics at the division plane (Lam et al., 2006). PopZ forms oligomeric networks at the poles to form polar ribosome exclusion zones and serves two roles during the cell cycle, switching function during the swarmer to stalk transition: it first interacts with the parS/ParB centromere at the stalked pole prior to the initiation of DNA replication, and later, during polar maturation, it recruits remodeling factors, such as peptidoglycan synthesis machinery, to transform the flagellated pole into a stalked pole (Bowman et al., 2010; Laloux and Jacobs-Wagner, 2013; Ptacin et al., 2014). All of these regulatory polar protein systems serve as examples of alternative networks that regulate the otherwise conserved core peptidoglycan synthesis machinery.

Another difference between *C. crescentus* and canonical rod-shaped bacteria is that it exhibits at least four peptidoglycan synthesis modes over its cell cycle: lateral elongation, elongation at the division plane before division (also called “pre-septal” or “medial” elongation, Figure 2F), septation, and stalk synthesis/elongation (Figure 4A). As in canonical rod-shaped cells, cell elongation in *C. crescentus* is MreB-dependent. Early studies showed that depletion of MreB disrupts cell morphology and results in lemon-shaped cells (Figge et al., 2004; Gitai et al., 2004). However, the role of MreB has expanded in *C. crescentus* to accommodate the cell cycle and additional growth modes. For example, MreB appears to influence cell polarity and division, as its depletion disturbs the localization of origins of replication as well as the localization of polar regulatory proteins PleC, DivJ, CckA, and DivK (Gitai et al., 2004). Moreover, MreB exhibits dynamic localization throughout the cell cycle, cycling between dispersed helical patterns in swarmer cells (similar to that seen in *E. coli* and *B. subtilis*) and an intense band at the division plane in pre-divisional cells (Figure 4B).

The dynamic localization of MreB drives a shift in growth mode from dispersed, lateral elongation mediated by MreB to zonal, medial elongation mediated by both MreB and FtsZ. The condensation of MreB at the division plane requires assembly of the FtsZ ring (Figge et al., 2004; Aaron et al., 2007), and high-resolution temporal studies have shown that MreB recruitment to the FtsZ ring coincides with medial elongation (Goley et al., 2011). *E. coli* also exhibits a transient pre-septal elongation mode (de Pedro et al., 1997; Aarsman et al., 2005; Potluri et al., 2012), in which FtsZ and MreB interact directly to transfer peptidoglycan synthesis machinery from the elongasome to the divisome (Fenton and Gerdes, 2013). However, in *C. crescentus,* this growth mode predominates a portion of the cell cycle. Because the FtsZ quickly reassembles after division in stalked cells, stalked cells likely elongate primarily via medial elongation (Aaron et al., 2007). Therefore, a similar medial elongation growth mode has been extended in *C. crescentus* to accommodate the asymmetric cell cycle.

MreB appears to have species-specific functions in *C. crescentus*. For example, it appears to interact directly or indirectly with CreS, and is required for proper attachment of the CreS filament to the membrane (Ausmees et al., 2003; Cabeen et al., 2009). Mutations along one surface of MreB alter cell curvature and disrupt the association of the CreS filament with the membrane (Charbon et al., 2009; Dye et al., 2011). Therefore MreB may organize CreS during cell growth, facilitating the attachment of the filament in a stretched conformation (Charbon et al., 2009). Another set of MreB mutations, which occur within the nucleotide-binding pocket, result in morphological defects and affect the localization pattern and dynamics of MreB (Dye et al., 2011). The nucleotide binding site mutants suggest that the ATP hydrolysis cycle of MreB could be coupled to the *C. crescentus* cell cycle to spatiotemporally regulate specific growth modes. The regulation of MreB by ATP could drive its relocation from the sides of the elongating cell to the division plane during the cell cycle.

Some MreB mutants have interesting implications for understanding how *C. crescentus* may remodel polar peptidoglycan. For a subset of the nucleotide binding site MreB mutants (E213G, D16G, N21D, and A325P), cells exhibit a variable width phenotype in which the ends of the cells become extremely tapered and pointed (Dye et al., 2011; Harris et al., 2014). In these mutants, although MreB still exhibits wild type function, it localizes to the cell poles instead of dispersing or condensing at the division plane (Harris et al., 2014). This behavior presumably drives aberrant peptidoglycan synthesis to create elongated cell poles. These MreB mutants may be trapped in a phase of the ATP cycle in which MreB localizes to the poles for a specific polar growth mode in wild type *C. crescentus* cells (Dye et al., 2011). Previous observations that the tapered shape of the wild type *C. crescentus* cell pole develops during the next cell cycle, not during septation or medial elongation (Aaron et al., 2007), corroborates the idea that MreB could participate in remodeling the cell wall at the poles. That the polar cell wall in *C. crescentus* undergoes remodeling instead of remaining entirely inert contradicts what is typically assumed for rod-shaped bacteria. Given that *C. crescentus* belongs to the Alphaproteobacteria class, many members of which exhibit polar growth (Brown et al., 2011, 2012), it may not be surprising if its poles do not follow the same rules as *E. coli* and *B. subtilis*.

In addition to its diversified polar regulators and various growth modes, *C. crescentus* hosts an expanded repertoire of bifunctional peptidoglycan synthases (penicillin-binding proteins, or PBPs). *E. coli* has three bifunctional PBPs. Although PBP1A and PBP1B exhibit semi-redundancy in function, PBP1A appears to preferentially associate with the elongasome (Banzhaf et al., 2012) and PBP1B with the divisome (Bertsche et al., 2006; Müller et al., 2007). The function of PBP1C remains unknown (Schiffer and Höltje, 1999). In contrast, *C. crescentus* has five predicted bifunctional PBPs (PBP1A, PbpC, PbpX, PbpY, PbpZ) which appear to be largely redundant, with any of them capable of functioning as the sole bifunctional PBP with the exception of PbpZ (Yakhnina and Gitai, 2013; Strobel et al., 2014). The same four enzymes were shown to be capable of interacting with divisome components FtsN, FtsL, and DipM (Strobel et al., 2014). Despite this redundancy, each PBP has a specific localization pattern during the cell cycle, suggesting specific cellular functions and roles: Pbp1A and PbpZ localize to the cell periphery; PbpY and PbpX to the periphery, division plane, and stalk; and PbpC to the stalk. Therefore, with the exception of PbpZ, these bifunctional PBPs have retained the ability to interact with both the elongasome and divisome while also evolving distinct affinities for specific localization factors.

*Caulobacter crescentus* exemplifies how the core peptidoglycan synthesis machinery can be adapted to create its specific morphology and to fit its lifestyle. While it retains an MreB-dependent elongasome and an FtsZ-dependent divisome, it has diversified its growth modes by adding what appears to be a dual MreB- and FtsZ-mediated zonal growth mode at the division plane. These growth modes are strictly regulated in order to synchronize them with the cell cycle and to accommodate asymmetric division. The vibrioid shape arises from diversifying cytoplasmic scaffolds (CreS) and changing the interacting partners of MreB to achieve cell cycle-dependent dynamics. In addition, *C. crescentus* has expanded its suite of bifunctional PBPs and may have additional peptidoglycan-modifying enzymes. Finally, *C. crescentus* has evolved a specialized growth mode, stalk synthesis, which is described in the next section.

## The Stalk as a Model System for Understanding Zonal Growth

Stalk synthesis and elongation constitutes one growth mode unique to *C. crescentus* among model rod-shaped bacteria (although not among Alphaproteobacteria). A thin extension of the inner membrane, peptidoglycan, and outer membrane layers (Poindexter, 1964), the stalk is a morphologically distinct organelle thought to improve the nutrient scavenging ability of the cell (Poindexter, 1978; Ireland et al., 2002; Wagner et al., 2006). The narrow cytoplasm of the stalk is free of DNA, ribosomes and most cytoplasmic proteins (Poindexter and Cohen-Bazire, 1964; Ireland et al., 2002; Wagner et al., 2006) and compartmentalized by cross-bands, disk-like, proteinaceous structures that intersect the width of the stalk perpendicular to the long axis of the cell (Jones and Schmidt, 1973; Schlimpert et al., 2012). Cross-bands prevent exchange of membrane and soluble proteins between the stalk and cell body (Schlimpert et al., 2012). As a nonessential organelle, the stalk serves as a convenient model for zonal, or targeted, peptidoglycan growth (Wagner and Brun, 2007).

Stalk synthesis initiates at the cell pole during the swarmer to stalked cell transition phase of the cell cycle, and elongation occurs at the cell-stalk junction during pre-divisional cell elongation (Schmidt and Stanier, 1966; Aaron et al., 2007). MreB and RodA (an MreB-associated protein) depletion, or treatment with the PBP2 inhibitor mecillinam (Seitz and Brun, 1998), result in the loss or shortening of stalks, and recovery from depletion leads to the growth of ectopic stalked poles, suggesting that MreB, in concert with RodA and a PBP2 homolog, plays a role in recruiting peptidoglycan synthesis machinery for stalk synthesis (Wagner et al., 2005). Overexpression of RodZ (another MreB-associated protein) resulted in multiple stalks forming at the same pole, opposite pole, or on the cell body (Alyahya et al., 2009). RodZ localization dynamics coincided with FtsZ during the cell cycle but appeared to depend on MreB. Overall, these data suggest that stalk synthesis and elongation in *C. crescentus* potentially depends on a similar core apparatus as elongation, utilizing MreB as a scaffold. The mechanism for cross-band formation may have interesting intersections with the processes of stalk synthesis and elongation. Early on, electron micrographs of *C. crescentus* stalks showed that cross-bands consisted of concentric circular striations, possibly of peptidoglycan and membranes (Jones and Schmidt, 1973). Because the stalk continues to elongate as the stalked cell participates in increasing rounds of division, it was postulated that stalk length and number of cross-bands could indicate cell age. Quantitative analysis suggested that one cross-band is added to the stalk toward the end of each reproductive cycle, possibly accompanying division (Poindexter and Staley, 1996; Schlimpert et al., 2012). The idea that cross-bands consist of peptidoglycan has been challenged by the discovery that cross-bands are the product of the macromolecular assembly of least four proteins, StpABCD (Schlimpert et al., 2012). However, a relationship between cross-band assembly and the peptidoglycan synthesis machinery may still exist.

Like septation, lateral, or medial elongation in *C. crescentus*, stalk synthesis and elongation appear to be highly tuned to cell cycle regulation and polar positioning mechanisms. Moreover, the mechanism for stalk synthesis appears to depend on the expansion and diversification of the core machinery. Bactofilins BacA and BacB constitute a class of scaffolding proteins with proline-rich terminal regions that localize to the stalked pole of the cell during the swarmer to stalked cell transition (Kühn et al., 2010). Deletion of these genes led to a 45% reduction in stalk length, suggesting a recruitment role in stalk assembly. Indeed, the localization of the stalk-specific bifunctional peptidoglycan synthase PbpC to the stalk was shown to be dependent on BacA and BacB. PbpC potentially acts in conjunction with PbpX in stalk elongation (Yakhnina and Gitai, 2013; Strobel et al., 2014), however, it has also been shown to have a role in recruiting and modifying a stalk-specific protein. StpX is a stalk-specific membrane protein that promotes the elongation of the stalk in nutrient-limiting conditions (Hughes et al., 2010). PbpC is not only required for localizing StpX to the stalk-cell junction at onset of stalk synthesis, but appears to be directly or indirectly involved in the process of tethering StpX to the outer membrane or to an outer membrane protein (Hughes et al., 2013). That this phenomenon takes place regardless of PbpC’s transglycosylase or transpeptidase activities suggests alternative roles for various PBPs and other enzyme classes in regulating the peptidoglycan synthesis machinery, opening up the diversity of mechanisms for the evolution of novel functions.

Phylogenic studies indicate that stalk positioning evolved from an ancestral, single polar stalk in the order *Caulobacteraceae*, to a single sub-polar stalk in *Asticcacaulis excentricus*, and subsequently to bilateral stalks in *Asticcacaulis biprosthecum* (Jiang et al., 2014). The stalk structure appears identical in *Caulobacter* and *Asticcacaulis* (Pate and Ordal, 1965) and all three species appear to share the same stalk synthesis mechanism of inserting peptidoglycan at the base of the stalk (Jiang et al., 2014). The natural variation and evolution of stalk positioning in these species correlates with the localization of SpmX, a developmental regulator (Radhakrishnan et al., 2008) that has been shown to be necessary for stalk synthesis in *Asticcacaulis* but not in *Caulobacter* (Radhakrishnan et al., 2008; Jiang et al., 2014). Therefore, in *Asticcacaulis* SpmX has been co-opted as a stalk-positioning factor. SpmX consists of three defined regions: a highly conserved N-terminal muramidase domain, an unstructured intermediate region that has been expanded by over 370 amino acids in the *Asticcacaulis* genus, and a two-pass transmembrane domain. Recent work has demonstrated that either the intermediate region or the transmembrane domain contain the residues responsible for SpmX’s new role in sub-polar and bilateral stalk positioning (Jiang et al., 2014). The muramidase domain, while necessary for overall function and localization, is interchangeable amongst species. How a murein hydrolase participates in SpmX’s function and localization will be an interesting question for future studies.

The recent discovery that SpmX coordinates stalk placement in the *Asticcacaulis* genus underscores the utility of not only studying the stalk as a model system for zonal growth, but of exploiting the natural diversity of stalked morphologies observed in Alphaproteobacteria. In marked contrast to division and elongation genes, which require depletion strains, stalk-less mutants are far easier to identify in a genetic screen. *Asticcacaulis* provides an opportunity for screening stalkless mutants with fewer chances of impacting elements critical for cell viability, since stalk synthesis no longer overlaps with polar regulation. Finally, altering the location of the stalk may prove to be a far more advantageous phenotype than losing the stalk entirely in terms of discovering localization and recruitment factors for stalk initiation and maintenance. Therefore studying stalk localization, initiation, and maintenance in *Asticcacaulis* will likely make significant inroads into understanding how bacteria can redirect the core peptidoglycan machinery to create novel morphologies.

## Conclusion and Summary

Critical advances in studying bacterial growth and division in model bacteria such as *E. coli*, *B. subtilis*, and *C. crescentus* have identified and characterized the fundamental components of the molecular machinery responsible for creating and shaping the sacculus. This expanding body of work reveals a general strategy for growing and shaping the bacterial cell wall. Moreover, it suggests ways in which the core can be adapted for various bacterial lifestyles as well as molecular mechanisms for the evolution of novel morphologies. The peptidoglycan synthesis machinery consists of a central core of cytoskeletal-like scaffolding proteins, inner membrane-spanning elements, and periplasmic or extracellular peptidoglycan-modifying elements. The modularity and evolvability of this system is inherent in how different elements have been retuned or repurposed in each genus for species-specific growth modes and morphologies. As different species of bacteria become genetically tractable, it is becoming clear that the diversification and expansion of any of the core elements of the peptidoglycan synthesis assembly underlie morphological diversity and alternate growth modes.

As studies in *C. crescentus* and the closely related genus *Asticcacaulis* demonstrate, much can be learned about general growth strategies using comparative approaches with related families. The great diversity of morphologies and growth modes in Alphaproteobacteria (Figure 4) offers an opportunity to exploit natural diversity to dissect mechanisms for stalk localization and synthesis, as well as polar growth. The recent advances in expressing fluorescent fusions in *Hyphomonas neptunium* open up yet another opportunity for discovering genes responsible for polar growth and stalk development (Jung et al., 2014). The increasing ease and affordability of genome sequencing allows further investigation into the suites of genes underlying uncharacterized growth modes in other genera. New reagents that fluorescently label sites of peptidoglycan synthesis are providing simple methods to determine growth modes, even in understudied species and environmental samples (Kuru et al., 2012; Pilhofer et al., 2013; Liechti et al., 2014). Determining how bacteria actively restructure their morphologies, or how they have evolved various morphologies over time, remains a major goal in bacteriology. Expanding investigations into understudied genera with novel morphologies and growth modes will complement and enrich our understanding of how bacteria grow and proliferate.

### Conflict of Interest Statement

The authors declare that the research was conducted in the absence of any commercial or financial relationships that could be construed as a potential conflict of interest.
